# A panel data set on harvest and perfusion decellularization of porcine rectus abdominis

**DOI:** 10.1016/j.dib.2016.04.018

**Published:** 2016-04-13

**Authors:** Jian Zhang, Wen Yue Cheng, Zhi Qian Hu, Neill J. Turner, Li Zhang, Qiang Wang, Stephen F. Badylak

**Affiliations:** a Department of Surgery, Shanghai Chang Zheng Hospital, Second Military Medical University, Shanghai 200003, PR China; b Department of Regenerative Medicine, Shanghai Zhabei District Central Hospital, Shanghai 200072, PR China; c McGowan Institute for Regenerative Medicine, University of Pittsburgh, Pittsburgh, PA 15219, USA; d Department of General Surgery, Shanghai Zhabei District Central Hospital, Shanghai 200072, PR China; e Department of Surgery, University of Pittsburgh, Pittsburgh, PA 15129, USA

## Abstract

In this dataset, we particularly depicted the harvest and perfusion decellularization of porcine rectus abdominis (RA), accompanied with displaying of the retained vascular trees within the perfusion-decellularized skeletal muscle matrix (pM-ECM) using vascular corrosion casting. In addition, several important tips for successful pM-ECM preparation were emphasized, which including using anatomically isolated skeletal muscle as tissue source with all main feeding and draining vessels perfused, preserving the internal microcirculation availability, aseptic technique and pyrogen free in all steps, sequential perfusion via artery or vein, and longtime washing after decellularization. The data are supplemental to our original research article describing detailed associations of pM-ECM as a clinically relevant scale, three-dimensional scaffold with a vascular network template for tissue-specific regeneration, “Perfusion-decellularized skeletal muscle as a three-dimensional scaffold with a vascular network template” Zhang et al. (2016) [1].

## Specification table

**Table:** 

Subject area	Biology

More specific subject area	Extracellular matrix scaffold preparation-regenerative medicine
Type of data	Text file, table, figure
How data was acquired	Canon 550D, surgical harvest, perfusion decellularization, vascular corrosion casting
Data format	Raw, processed
Experimental factors	No pre-treatment
Experimental features	Catheterization and perfusion of porcine RA before harvest, Harvest of inferior porcine RA, Sample preparation for perfusion decellularization, Perfusion decellularization of porcine RA, Macroscopic check, dye infusion assessment and vascular corrosion casting
Data source location	Second Military Medical University, Shanghai, China, Shanghai Zhabei District Central Hospital, Shanghai, China, McGowan Institute for Regenerative Medicine, University of Pittsburgh, Pittsburgh, USA
Data accessibility	The data are with this article.

## Value of the data

● This data will be helpful for the research community that harvests and decellularizes porcine rectus abdominis (RA) via the vasculature perfusion for regenerative medicine and tissue engineering.
● This data allows the scientific community to prepare RA pM-ECM on a clinically relevant scale with similar shape and huge volume to human skeletal muscle.
● This data elucidates the unique retained vascular trees in pM-ECM using corrosion casting.
● This data provides the research community to gain more insight about perfusion decellularization for biologic graft from solid organ with higher density.

## 1 Data

**Fig. 1:**
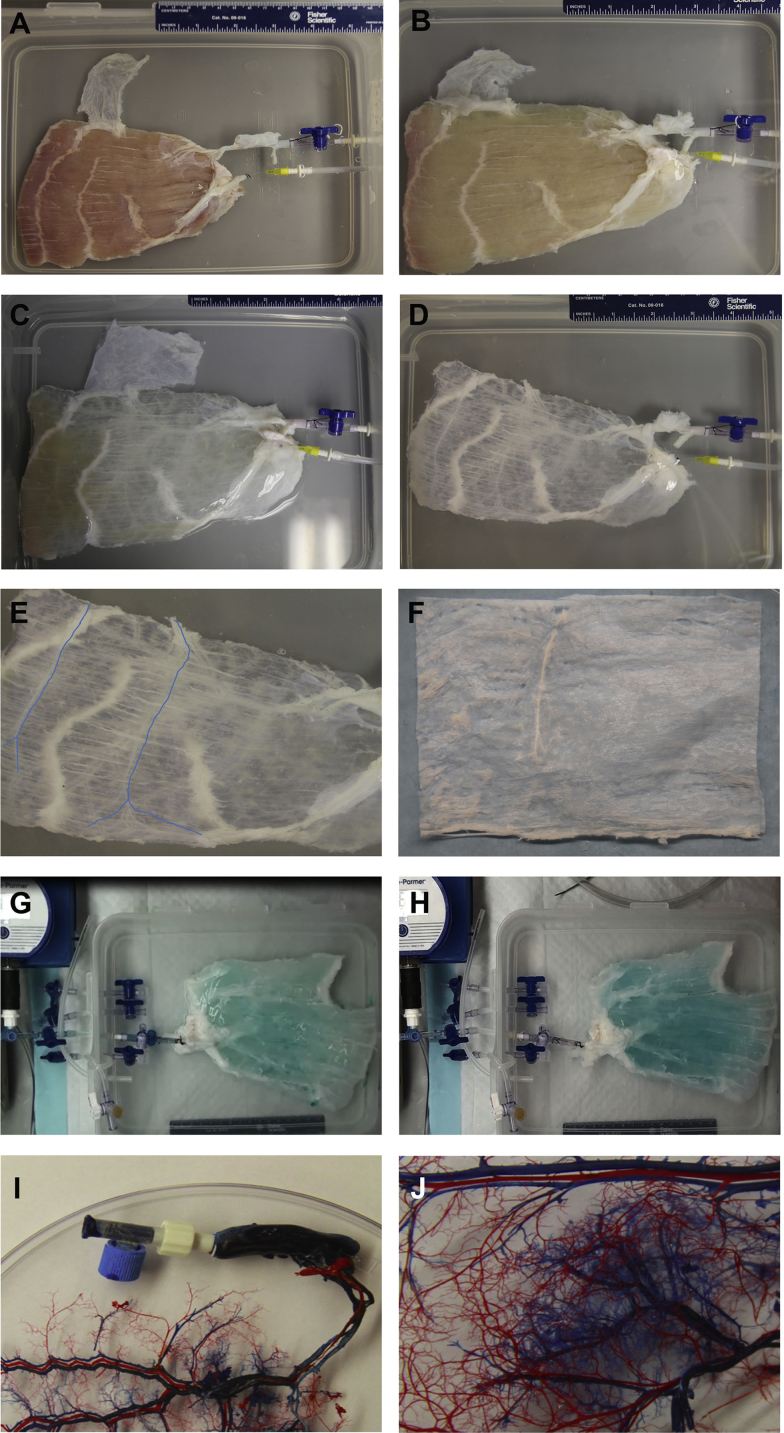
Perfusion decellularization of the porcine RA and macroscopic observation of pM-ECM. Representative gross appearance of the surgical harvested inferior half RA before decellularization (A) and immediately after trypsin (B), sodium dodecyl sulfate (C), Triton-X 100 treatment (D). (E) The lateral neural pathway was retained in the pM-ECM (blue trend line). (F) The resulting pM-ECM sheet. (G and H) The pM-ECM maintained its shape and size to the original native muscle tissue, and kept the structural integrity. (I and J) Arterial (red) and venous (blue) corrosion casts study confirming the preservation of large vessels and their extensive network of microvascular branches.

Fig. 1 shows the gross appearance of the surgical harvested inferior half RA before decellularization (Fig. 1A) and immediately after consequent perfusion with trypsin (Fig.1B), sodium dodecyl sulfate (SDS) (Fig. 1C) and Triton-X 100 (Fig. 1D). The lateral neural pathway was retained in the pM-ECM (blue trend line, Fig. 1E). Fig. 1F displays the sliced pM-ECM. The pM-ECM maintained its shape and size to the original native muscle tissue, and kept the structural integrity (Fig. 1G and H). Arterial (red) and venous (blue) corrosion casting study confirms the preservation of large vessels and their extensive network of microvascular branches (Fig. 1I and J). The baseline characteristics and the biologic potential of the RA pM-ECM as a scaffold for supporting site appropriate, tissue reconstruction have been described previously [1]. Comparatively, the pM-ECM preparation is of higher technology difficulty than the other whole-organ, or sub-organ perfusion-decellularizion [2], [3], [4], [5], [6], [7], [8], [9], [10], [11], [12], [13], and non-perfusion skeletal muscle sheet decellularizion [14], [15], [16], [17].

**Fig. 2:**
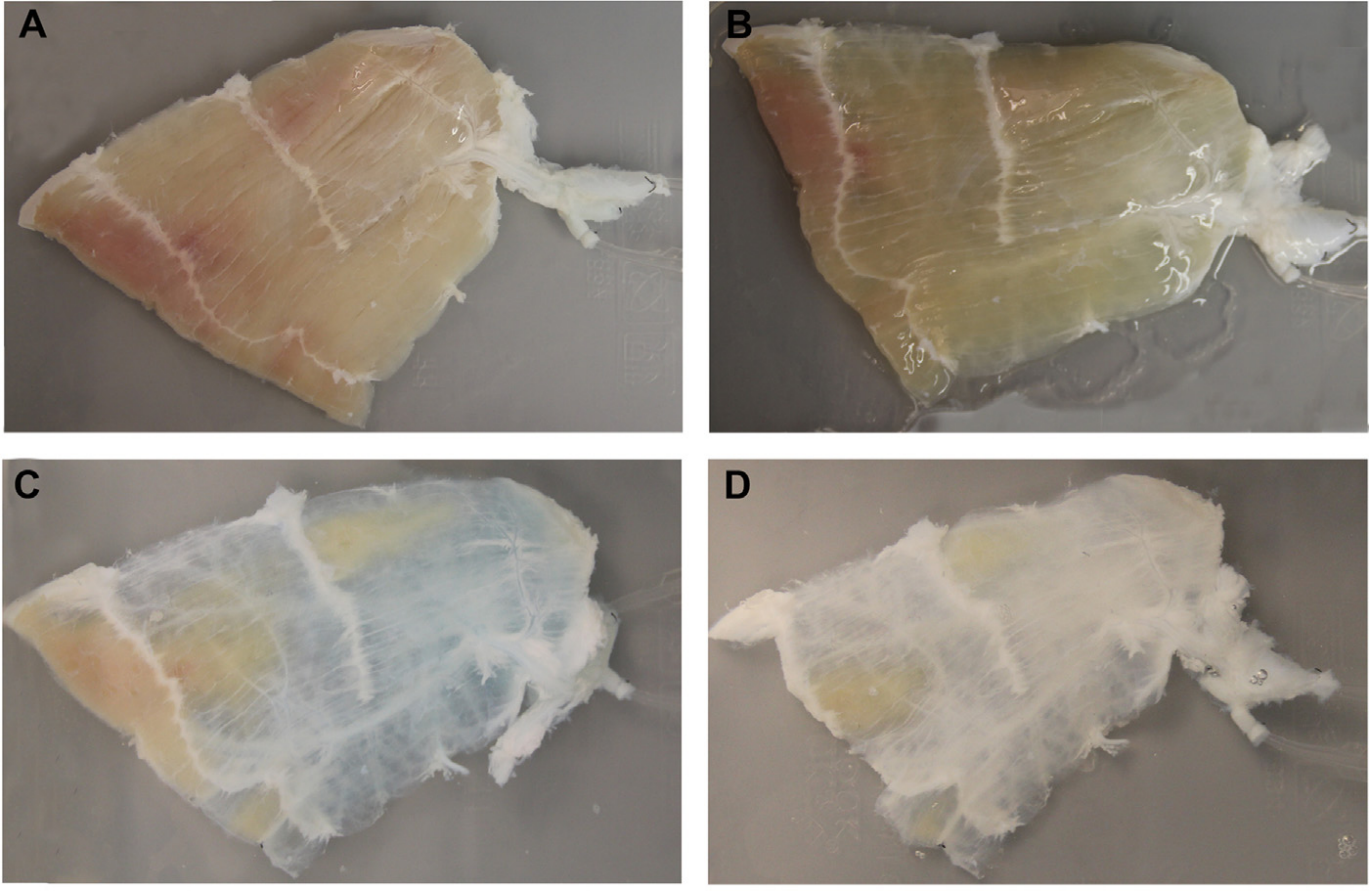
Macroscopic appearance of an unsuccessful perfusion decellularization of the porcine RA due to the internal microcirculation unavailability. Blood colored sample parts would not get decellularized even with higher detergent concentration and longer perfusion time. (A) Saline perfusion before decellularization. (B) 1% SDS perfusion for 24 h. (C) 3% Triton-X 100 perfusion for 24 h. (D) 3% Triton-X 100 perfusion for 72 h.

Fig. 2 depicts the invalid perfused RA due to the internal microcirculation unavailability.

**Fig. 3:**
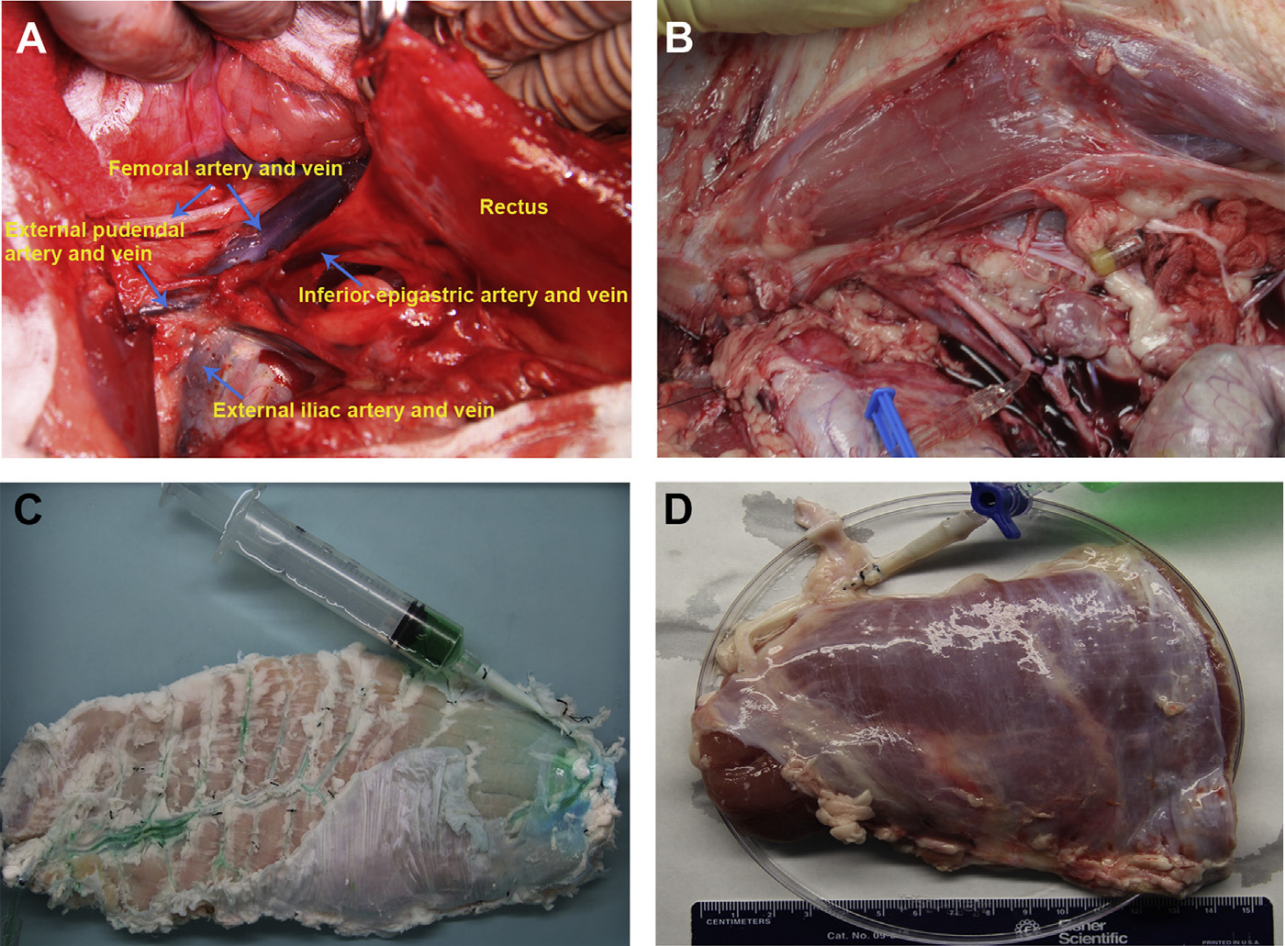
Harvest of porcine RA. (A) The external iliac artery, femoral artery and pudendal epigastricus trunk, which is formed by inferior epigastric artery, epigastric caudalis superficialis artery and pudenda external artery, together with their accompanying veins were carefully isolated and skeletonized. (B) Antegrade perfusion via inferior epigastric artery was performed prior to harvesting of RA. (C) The harvested RA was trimmed of adherent adipose and connective tissues, and vascular dye perfusion was performed to conform to the preserving vascular network integrity. (D) The harvested distal half of RA sample ready for further perfusion decellularization.

Fig. 3 shows the harvest of porcine RA.

Table 1 summarizes the steps in the perfusion preparation of pM-ECM.

## 2 Experimental design, materials and methods

### 2.1 Porcine RA retrieval

Adult female Yorkshire pigs (24 week old, mean 75 kg in weight) were used for the RA retrieval. The animals were sedated 30 min before induction of anesthesia with an intramuscular injection of azaperone (4 mg/kg), followed by intramuscular injection of ketamine (10 mg/kg) and midazolam (1 mg/kg). Following systemic heparinization at a dose of 10000UI per animal administrated through the auricular marginal vein, whole RA (right or left) below the umbilical point and above the pubic symphysis and the pubic bone crest was collected through a long extra-peritoneal midline incision under sterile conditions. The external iliac artery, the femoral artery and the pudendal epigastricus trunk, which is formed by inferior epigastric artery, epigastric caudalis superficialis artery and pudenda external artery, together with their accompanying veins were carefully isolated and skeletonized, after pulling the peritoneum cranially with partial exposure of the retroperitoneal space (Fig. 3A). The RA was then infused with 0.9% saline with 50UI/ml heparin through the external iliac artery and the inferior epigastric artery at a speed of 30–50 ml/min by a pump for 5 min. The intra-arterial pressure was maintained at 110–150 mmHg during infusion (Fig. 3B). As soon as the RA turned white and infused liquid flowing out from veins became colorless, the RA was then harvested by detaching the posterior sheath from the peritoneum and transverse abdominis, dissecting along the surface of anterior sheath, and transecting all the anterior perforating branches to external, internal oblique muscle and all the posterior perforating branches to transverse muscle as well as the posterior perforating vessels to the inferior border of costal arch. The RA was then carefully dissected free from that platysmal aponeurosis along the linea semilunaris. The perforator vessels on the RA surface were identified and ligated separately with surgical threads (Fig. 3C). In order to make it easier for catheterization of inferior epigastric vessels and more feasible for the expected graft-vascular anastomosis, an ideal vascular cutoff point at the external iliac artery and vein should be considered. After harvest, the inferior epigastric pedicle was catheterized with i.v. needles (venous-18 G; arterial-22 G) and the samples were stored in 0.9% saline with 50 UI/ml heparin surrounded by ice in the container during transportation to the lab. The distal half of RA was chosen because of its similarities with humans in terms of fewer perforator vessels and linea transversae compared with upper part of RA (Fig. 3D).

### 2.2 Perfusion decellularization

After cleaned the attached fat and connective tissue under saline perfusion, the obtained porcine distal RA was put in a sterilized bioreactor and decellularized by continuous perfusion using a series of chemical and enzymatic treatments via the inferior epigastric artery and vein in a perfusion bioreactor (Table 1). Briefly, 0.02% Trypsin/0.05% ethylene glycol-bis-(β-amino-ethylether) N, N, N′, N′-tetra-acetic acid (EGTA) (at 37 °C, pH=7.8) was infused via the artery for 1.75 h followed by 0.25 h via the vein. This was followed by 12 h perfusion (11 h via artery and 1 h via vein) of 0.1% SDS (Sigma, St Louis MO) in deionized water, 12 h perfusion (11 h via artery and 1 h via vein) of 1% Triton-X 100 (Sigma), a 2 h perfusion via artery of 0.1% PAA/4% ethanol (ETOH) and a 0.5 h perfusion via artery of DNase (40 U/ml, Sigma)/α-galactosidase (10 U/ml, Sigma). The next step involved extensive perfusion with sterilized deionized water, via the artery for up to 7 days to remove any residual detergent. The perfusion speed was adjusted according to that the perfusion pressure within supporting artery was maintained at 110–150 mmHg, usually ranging from 8–20 ml/min, and 1–2 mmHg in vein, usually no more than 1 ml/min. The resultant pM-ECM scaffolds were kept in sterilized 1×PBS solution, or lyophilized, or sliced (Sirman Palladio 300, Italy) and/or powdered (Mill Mini 3383L40, Thomas Wiley) for further analysis.

### 2.3 Implementation tips for RA harvest and perfusion decellularization

We emphasized several important tips for successful preparation of pM-ECM, including using anatomically isolated skeletal muscle as tissue source with protection and perfusion of all main feeding and draining vessels involved, preserving the internal microcirculation availability, aseptic and pyrogen free techniques in all steps, sequential perfusion via artery or vein, and longtime washing after decellularization. It should maintain the internal microcirculation availability and prevent myoglobin degradation and microcirculation occlusion during harvesting. Due to the internal microcirculation unavailability, the blood colored parts in RA would not get decellularized even with higher detergent concentration and longer perfusion time (Fig. 2A–D). Therefore, an unsatisfactory decellularization could be judged initially by appearance of this kind of invalid perfusion in RA. Secondly, as most muscle groups tend to have multiple blood supply, it is necessary to inset catheter into each main blood vessel to confirm blood supply. Thirdly, all the items exposed to pM-ECM, including bioreactor container, perfusion tube, catheter and needle, deionized water and detergent solution, should be strictly sterilized and pyrogen free. The amphipathic nature of lipopolysaccharide (LPS) contributes to its affinity for products with hydrophobic moieties inside pM-ECM. If the pM-ECM were contaminated with bacteria, we should be totally in a very difficult position to get rid of LPS by sterilization with PAA perfusion and thorough deionized water washing. Fourthly, perfusion sequence via artery or vein and longtime washing after decellularization are strict for DNA and residual detergent clearance, and we should avoid perfusing artery and vein simultaneously.

### 2.4 Integrity check

Under video surveillance, the dye solution was continuously perfused into the pM-ECM via artery. With raising of the perfusion pressure to 300 mmHg, scaffold integrity in pM-ECM will be confirmed in absence of dye leakage.

### 2.5 Vascular corrosion casts

Vascular corrosion casting was carried out for graft vasculature using Batson׳s No. 17 Plastic Replica and Corrosion Kit (Polysciences, Inc.) according to the instructions. The kit consists of partially polymerized monomer, catalyst, and promoter to allow curing at room temperature after injection, with red and blue pigments supplying for contrast. We simultaneously displayed the retained artery as red and vein as blue within the pM-ECM. The pigments (0.5 g red or blue) were added to 25 ml base solution relatively prior to mixing with the catalyst and promoter. Stirred vigorously with a spatula until mixed together thoroughly and then divided into two equal parts (12.5 ml relatively). Carefully add 4 ml of the catalyst to 12.5 ml of base solution. Set aside until the second half is mixed. Carefully add 3 drops of promoter to the second half of the base solution and mix slowly on a magnetic stirrer. Added the two solutions together and stirred to mix. The injections were made from external iliac artery and vein with a disposable polyethylene syringe within 30–45 min. The injected specimen was fully cured in 2–3 h. It is preferable to keep the specimen in cold water or ice bath during the curing process to aid in the dissipation of the exothermic reaction caused by polymerization. Controlling the temperature will prevent expansion and distortion of the specimen. After fully curing, the specimen is placed in the maceration solution (Catalog no. 07359) at 50 °C to corrode. The amount of solution should be at least 2–3 times the volume of the mass to be macerated. The specimen was removed every 2–3 h and rinsed in water to remove the excess material and allow the fluid to penetrate additional tissue. After 3 h, the solution became very cloudy with debris floating, the solution was changed. Scaffold sample was removed in 6 h.

## Figures and Tables

**Table 1:** Summary of the steps in the perfusion preparation of pM-ECM.

Chemical	Perfusion vessel	Length of treatment (h)
0.02% Trypsin/0.05% EGTA	Artery	1.75
Vein	0.25
Deionized water	Artery	0.50
2×PBS	Artery	0.50
0.1% SDS	Artery	11.00
Vein	1.00
Deionized water	Artery	0.50
Vein	0.50
1% Triton-X 100	Artery	11.00
Vein	1.00
Deionized water	Artery	0.50
Vein	0.50
0.1% PAA/4% ETOH	Artery	2.00
Deionized water	Artery	0.50
DNase/α- galactosidase	Artery	0.50
Deionized water	Artery	72.00

## References

[1] Zhang J., Hu Z.Q., Turner N.J., Teng S.F., Cheng W.Y., Zhou H.Y., Zhang L., Hu H.W., Wang Q., Badylak S.F. (2016). Perfusion-decellularized skeletal muscle as a three-dimensional scaffold with a vascular network template. Biomaterials.

[2] Ott H.C., Matthiesen T.S., Goh S.K., Black L.D., Kren S.M., Netoff T.I., Taylor D.A. (2008). Perfusion-decellularized matrix: using nature׳s platform to engineer a bioartificial heart. Nat. Med..

[3] Uygun B.E., Soto-Gutierrez A., Yagi H., Izamis M.L., Guzzardi M.A., Shulman C., Milwid J., Kobayashi N., Tilles A., Berthiaume F., Hertl M., Nahmias Y., Yarmush M.L., Uygun K. (2010). Organ reengineering through development of a transplantable recellularized liver graft using decellularized liver matrix. Nat. Med..

[4] Baptista P.M., Siddiqui M.M., Lozier G., Rodriguez S.R., Atala A., Soker S. (2011). The use of whole organ decellularization for the generation of a vascularized liver organoid. Hepatology.

[5] Soto-Gutierrez A., Zhang L., Medberry C., Fukumitsu K., Faulk D., Jiang H., Reing J., Gramignoli R., Komori J., Ross M., Nagaya M., Lagasse E., Stolz D., Strom S.C., Fox I.J., Badylak S.F. (2011). A whole-organ regenerative medicine approach for liver replacement. Tissue Eng. Part C Methods.

[6] Petersen T.H., Calle E.A., Zhao L., Lee E.J., Gui L., Raredon M.B., Gavrilov K., Yi T., Zhuang Z.W., Breuer C., Herzog E., Niklason L.E. (2010). Tissue-engineered lungs for in vivo implantation. Science.

[7] Ott H.C., Clippinger B., Conrad C., Schuetz C., Pomerantseva I., Ikonomou L., Kotton L.D., Vacanti J.P. (2010). Regeneration and orthotopic transplantation of a bioartificial lung. Nat. Med..

[8] Orlando G., Farney A.C., Iskandar S.S., Mirmalek-Sani S.H., Sullivan D.C., Moran E., AbouShwareb T., De Coppi P., Wood K.J., Stratta R.J., Atala A., Yoo J.J., Soker S. (2012). Production and implantation of renal extracellular matrix scaffolds from porcine kidneys as a platform for renal bioengineering investigations. Ann. Surg..

[9] Sullivan D.C., Mirmalek-Sani S.H., Deegan D.B., Baptista P.M., Aboushwareb T., Atala A., Yoo J.J. (2012). Decellularization methods of porcine kidneys for whole organ engineering using a high-throughput system. Biomaterials.

[10] Goh S.K., Bertera S., Olsen P., Candiello J.E., Halfter W., Uechi G., Balasubramani M., Johnson S.A., Sicari B.M., Kollar E., Badylak S.F., Banerjee I. (2013). Perfusion-decellularized pancreas as a natural 3D scaffold for pancreatic tissue and whole organ engineering. Biomaterials.

[11] Jank B.J., Xiong L., Moser P.T., Guyette J.P., Ren X., Cetrulo C.L., Leonard D.A., Fernandez L., Fagan S.P., Ott H.C. (2015). Engineered composite tissue as a bioartificial limb graft. Biomaterials.

[12] Mertsching H., Schanz J., Steger V., Schandar M., Schenk M., Hansmann J., Dally I., Friedel G., Walles T. (2009). Generation and transplantation of an autologous vascularized bioartificial human tissue. Transplantation.

[13] Mertsching H., Walles T., Hofmann M., Schanz J., Knapp W.H. (2005). Engineering of a vascularized scaffold for artificial tissue and organ generation. Biomaterials.

[14] Perniconi B., Costa A., Aulino P., Teodori L., Adamo S., Coletti D. (2011). The pro-myogenic environment provided by whole organ scale acellular scaffolds from skeletal muscle. Biomaterials.

[15] Wolf M.T., Daly K.A., Reing J.E., Badylak S.F. (2012). Biologic scaffold composed of skeletal muscle extracellular matrix. Biomaterials.

[16] Wang L., Johnson J.A., Chang D.W., Zhang Q. (2013). Decellularized musculofascial extracellular matrix for tissue engineering. Biomaterials.

[17] Porzionato A., Sfriso M.M., Pontini A., Macchi V., Petrelli L., Pavan P.G., Natali A.N., Bassetto F., Vindigni V., De Caro R. (2015). Decellularized human skeletal muscle as biologic scaffold for reconstructive surgery. Int. J. Mol. Sci..

